# Supplementary material for: The Personalized Nutrition Study (POINTS): evaluation of a genetically informed weight loss approach, a Randomized Clinical Trial
Source: Nat Commun. 2023 Oct 9;14:6321. doi: 10.1038/s41467-023-41969-1 (PMC10562431; doi:10.1038/s41467-023-41969-1)
Supplement: Supplementary file 1 — Supplementary Information [file 41467_2023_41969_MOESM1_ESM.pdf]

SUPPLEMENTARY INFORMATION

The Personalized Nutrition Study (POINTS): Evaluation of a genetically informed weight loss approach. A Randomized Clinical Trial.

TABLE OF CONTENTS

SUPPLEMENTARY METHODS 2
- **Genotype scoring criteria** 2
  - *Original scoring form* 2
  - ***Supplementary Table 1*** 3
  - *Updated scoring form* 3
  - ***Supplementary Table 2*** 4
- **Intervention sessions and delivery** 5
- **Outcome materials** 5
  - *Appetitive traits, food cravings, and food preferences* 5
  - *Diet personalization* 6
  - *Intervention satisfaction* 6

SUPPLEMENTARY RESULTS 7
- **Supplementary Table 3** 7
- **Supplementary Table 4** 8
- **Supplementary Table 5** 10
- **Supplementary Table 6** 12
- **Supplementary Table 7** 14
- **Supplementary Table 8** 16
- **Supplementary Figure 1** 19
- **Supplementary Figure 2** 20
- **Supplementary Table 9** 21

SUPPLEMENTARY REFERENCES 22

SUPPLEMENTARY METHODS

Genotype scoring criteria

Participants were identified as carbohydrate- and fat-responders *a priori* based on their combined genotypes at multiple genetic variants. The genetic information was accessed via the raw data from each participant's genealogy test. If participants had completed a genealogy test before the study and were able to share that information with the study staff, their data was reviewed to verify eligibility. If participants had not completed a genealogy test before the study, they were provided with a genealogy test kit, and the data was reviewed as soon as they were available to determine eligibility.

Original scoring form

Appropriate participant enrollment initially required the genotypes for 6 single nucleotide polymorphisms (SNPs). The 6 SNPs considered were:

1. *FGF21rs838147*
2. *TCF7L2rs12255372*
3. *IRS1rs2943641*
4. *APOA5rs662799*
5. *PLIN1rs894160*
6. *APOA2rs5082*

Every SNP had a 'responsive' genotype. These genotypes determined whether participants were predicted fat-responders (predicted to lose more weight on a high-fat/ low-carbohydrate diet), predicted carbohydrate-responders (predicted to lose more weight on a high-carbohydrate/ low-fat diet), or if they were ineligible (responsive genotypes for neither or both diets). The 'responsive' genotypes for each SNP are illustrated in **Supplementary Table 1**.

Supplementary Table 1. Each SNP's 'responsive' genotype and the related diet

SNP	High-fat/ low-carbohydrate	High-carbohydrate/ low-fat
FGF21rs838147	TT	CC
TCF7L2rs12255372	-	TT
IRS1rs2943641	-	CC
APOA5rs662799	CC, CT/TC	TT
PLIN1rs894160	GG	AA
APOA2rs5082	-	CC

Participants were classified as a predicted fat-responder or carbohydrate-responder if they had one or more of the 'responsive' genotypes for a specified diet (high-fat/ low-carbohydrate vs high-carbohydrate/ low-fat) and if they did not have any of the 'responsive' genotypes for the alternative diet (**Supplementary Table 1**). Participants with 'responsive' genotypes for both or neither of the specified diets were excluded.

Updated scoring form

In the updated scoring criteria, appropriate participant enrolment required the genotypes for the following 10 SNPs:

1. *FGF21rs838147*
2. *TCF7L2rs12255372*
3. *IRS1rs2943641*
4. *APOA5rs662799*
5. *PLIN1rs894160*
6. *APOA2rs5082*
7. *FTOrs9939609*
8. *PPARGrs1801282*
9. *GIPRrs10423928*
10. *GYS2rs1478290*

For each genotype, scores ranging from 0-2 were given to predicted fat-responders (predicted to lose more weight on a high-fat/ low-carbohydrate diet) and predicted carbohydrate-responders (predicted to lose more weight on a high-carbohydrate/ low-fat diet), as illustrated in **Supplementary Table 2**. In case the raw data from the test kits did not provide the *a priori*-determined SNPs, highly correlated proxy SNPs were used.

Supplementary Table 2. Each SNP used for scoring criteria and the scores provided.

SNP	High-fat/ low-carbohydrate	High-carbohydrate/ low-fat
FGF21rs838147	CC=0, TC=1, TT=2	TT=0, TC=1, CC=2
TCF7L2rs12255372		GG=0, GT=0, TT=2
IRS1rs2943641		TT=0, TC=1, CC=2
APOA5rs662799	TT=0, TC=1, CC=2	CC=0, TC=1, TT=2
PLIN1rs894160	AA=0, GA=1, GG=2	GG=0, GA=1, AA=2
APOA2rs5082		TT=0, TC=0, CC=2
FTOrs9939609		TT=0, AT=1, AA=2
PPARGrs1800437 (proxy for *GIPRrs10423928*)	GG=0, CG=1, CC=2	CC=0, CG=1, GG=2
GIPRrs10423928	TT=0, AT=1, AA=2	
GYS2rs1478290	CC=0, AC=1, AA=2	

For example, the *APOA2rs5082* variant was included to identify those who may be responsive to changes in dietary fat based on previously available information.[1–5] The combined evidence from those studies supports that carriers of the CC genotype would respond to lowering dietary fat by losing more weight than those carrying either the CT or the TT genotype. Therefore, CC was given a score of “2” for responsiveness, whereas CT or TT scored “0”. A similar approach was taken for each of the SNPs included in the algorithm.

The aggregate scores for the high-fat/ low-carbohydrate and high-carbohydrate/ low-fat columns were calculated. Participants were then classified as a fat-responder if they scored ≥4 for high-

fat/ low-carbohydrate and <7 for high-carbohydrate/ low-fat, respectively. They were classified as carbohydrate-responder if they scored <4 for high-fat/ low-carbohydrate and ≥7 for high-carbohydrate/ low-fat, respectively. Participants fitting neither criterion were deemed ineligible.

Intervention sessions and delivery

The 12 weekly intervention sessions were tailored to the assigned diet and focused on (1) meal planning, (2) carbohydrates, (3) fats, (4) protein, (5) food labels, (6) fast food, (7) snacking and stress eating, (8) grocery shopping, (9) behavior changes (10) social support, (11) cravings and habits, and (12) special events. Intervention delivery was planned as in-person group sessions; however, due to the COVID-19 pandemic, most sessions were delivered remotely via webinar. Efforts were made for the first session to be in person, and overall, 78% of the sessions were virtual group sessions, and 22% were individual sessions.

Outcome materials

Appetitive traits, food cravings, and food preferences

Appetitive traits were measured with the Eating Inventory (EI),[6] food cravings were measured with the Food Craving Inventory (FCI)[7], and hedonic food preferences were measured with the Food Preference Questionnaire (FPQ)[8] at the W0 and W12 visits.

The EI is a 51-item measure of cognitive restraint, disinhibition (i.e., the tendency to overeat), and hunger that has been validated and is commonly used to assess eating behaviors in the fields of obesity and ingestive behavior research.[6]

The FCI measures general cravings for 33 different foods via a 5-point Likert scale from 1=never to 5=always. The instrument produces a total cravings score and scores reflecting cravings for high-fat foods, carbohydrates and starches, sweets, fast-food fats, and fruits and vegetables.[7]

The FPQ assesses hedonic preferences for 72 food items via a 9-point Likert scale from 1=dislike extremely to 9=like extremely. The food items are grouped into the following six food categories (12 foods per category): High fat/high simple sugar (HF/HS), low fat/high simple sugar (LF/HS), high fat/high complex carbohydrate (HF/HCCHO), low fat/high complex carbohydrate (LF/HCCHO), high fat/low carbohydrate/high protein (HF/LCHO/HP), and low fat/low carbohydrate/high protein (LF/LCHO/HP).[8]

Diet personalization

The Diet Personalization Survey was completed at the W0 and W12 visits and during the intervention session at W6. Via five 8-point Likert scale items (1=not at all; 8=very much), the survey assesses to what extent (1) participants believe the assigned diet fits their typical eating habits, (2) participants believe the assigned diet fits their lifestyle, and (3) participants believe they can lose weight more easily on the assigned diet. Further, the survey assesses how confident participants are (4) that they can successfully lose weight on the assigned diet and (5) that they can follow it.

Intervention satisfaction

The Intervention Satisfaction Survey was completed at the W12 visit, assessing participants' satisfaction with the intervention format via eight 8-point Likert scale items (1=not at all; 8=very much). Specifically, the instrument assessed satisfaction with (1) the group format, (2) the degree of support from the interventionist, (3) the intervention materials, (4) the support from other participants, (5) the amount of food in the meal plan, (6) the macronutrient content (i.e., amount of fat, carbohydrates, and protein) of the meal plan, (7) the personal progress towards weight management, and (8) the degree to which the diet helped manage hunger.

SUPPLEMENTARY RESULTS

Supplementary Table 3. Participant characteristics between completers (included in main analysis) and non-completers.

	**All participants (N=138)**	**Completers (n=122)**	**Non-Completers (n=16)**
Race, n (%)			
White	95 (66.7)	83 (68.0)	9 (56.2)
Black/ African American	41 (29.7)	36 (29.5)	5 (31.2)
Other	5 (3.6)	3 (2.5)	2 (12.5)
Sex, n (%)			
Female	114 (82.6)	102 (83.6)	12 (75.0)
Male	24 (17.4)	20 (16.4)	4 (25.0)
	**Mean (SD)**	**Mean (SD)**	**Mean (SD)**
Age, years	54.0 (13.3)	54.4 (13.2)	50.9 (13.7)
Weight, kg	95.4 (15.6)	94.3 (15.2)	104.1 (16.7)
BMI, kg/m^2	35.2 (5.2)	34.9 (5.1)	37.6 (5.8)
Body fat, %	45.2 (9.2)	45.0 (9.3)	46.7 (8.9)
Waist circumference, cm	109.7 (12.7)	109.0 (12.2)	114.7 (15.0)
Hip circumference, cm	119.5 (12.2)	118.9 (12.2)	123.7 (11.9)
Waist-Hip Ratio	0.92 (0.08)	0.92 (0.08)	0.92 (0.11)
SBP, mmHg	121.6 (11.9)	121.7 (11.9)	120.66 (12.5)
DBP, mmHg	75.4 (7.5)	74.7 (7.4)	80.4 (6.5)

Data are mean (SD) for continuous and n (%) for categorical variables.

Abbreviations: BMI, body mass index; DBP, diastolic blood pressure; SBP, systolic blood pressure; SD, standard deviation.

Supplementary Table 4. Baseline scores in appetitive traits, food cravings, food preferences, and diet personalization.

	Fat-responders (n=85)		Carbohydrate-responders (n=37)	
	High-fat diet (n=44)	High-carbohydrate diet (n=41)	High-fat diet (n=21)	High-carbohydrate diet (n=16)
	**Mean (SD)**	**Mean (SD)**	**Mean (SD)**	**Mean (SD)**
**Eating Inventory**				
Cognitive restraint	11.2 (4.4)	11.2 (4.1)	12.7 (3.5)	11.4 (5.0)
Disinhibition	8.4 (3.8)	8.9 (3.5)	8.3 (3.5)	7.7 (3.3)
Hunger	5.2 (3.3)	5.6 (3.3)	4.4 (2.8)	3.4 (3.0)
**Food Craving Inventory**				
High fats	2.3 (0.7)	2.4 (0.7)	2.5 (0.5)	2.1 (0.6)
Sweets	2.6 (0.8)	2.5 (0.8)	2.5 (0.7)	2.2 (0.7)
Carbohydrates/Starches	2.5 (0.8)	2.4 (0.7)	2.5 (0.6)	2.3 (0.4)
Fast-food fats	2.9 (0.8)	2.9 (0.7)	3.1 (0.7)	2.8 (0.4)
Fruits and vegetables	2.7 (0.8)	2.8 (0.8)	2.7 (0.7)	2.6 (0.6)
Total cravings	2.6 (0.6)	2.5 (0.6)	2.7 (0.4)	2.3 (0.4)
**Food Preference Questionnaire**				
HF/HS	5.5 (1.8)	5.4 (1.5)	5.1 (1.9)	4.4 (1.9)
LF/HS	5.4 (1.3)	5.5 (1.1)	5.7 (1.1)	5.1 (1.6)
HF/HCCHO	5.1 (1.6)	5.0 (1.3)	5.0 (1.7)	4.5 (1.2)
LF/HCCHO	4.9 (1.3)	5.3 (1.1)	5.2 (1.4)	5.2 (1.4)
HF/LCHO/HP	6.1 (1.5)	6.6 (1.3)	6.3 (1.2)	5.6 (1.3)
LF/LCHO/HP	5.4 (1.3)	5.6 (1.2)	5.7 (1.2)	5.5 (1.0)

**Diet Personalization Survey**				
The assigned diet…				
… fits my typical eating habits	4.9 (2.1)	4.4 (2.1)	5.0 (2.0)	4.8 (2.5)
… fits my lifestyle	5.3 (2.0)	4.9 (2.1)	5.5 (2.1)	4.9 (2.6)
… makes it easier to lose weight	6.6 (1.8)	6.2 (1.7)	6.6 (1.6)	5.9 (2.3)
I am confident that I can…				
… successfully lose weight on the assigned diet	6.9 (1.3)	6.7 (1.4)	6.7 (1.6)	6.2 (2.3)
… follow the assigned diet	6.7 (1.3)	6.4 (1.4)	7.4 (0.9)	6.6 (1.9)

Data are mean (SD) for continuous and n (%) for categorical variables.

Abbreviations: HF/HS, high fat/high simple sugar; LF/HS, low fat/high simple sugar; HF/HCCHO, high fat/high complex carbohydrate; LF/HCCHO, low fat/high complex carbohydrate; HF/LCHO/HP, high fat/low carbohydrate/high protein; LF/LCHO/HP, low fat/low carbohydrate/high protein; SD, standard deviation.

Supplementary Table 5. Change in weight (kg and %), percent body fat, body composition, and blood pressure during the 12-week intervention in those assigned to a diet concordant vs. discordant with the genotype (including raw difference).

All participants	**Genotype-concordant diet (n=60)**		**Genotype-discordant diet (n=62)**				
	**Mean**	**(SD)**	**Mean**	**(SD)**	**Raw difference (95% CI)**		***p*-value**
Weight change, kg	−5.3	(0.5)	−4.7	(0.5)	−0.6	(−2.1, 0.9)	0.422
Weight change, %	−5.7	(0.6)	−5.2	(0.6)	−0.6	(−2.2, 1.1)	0.495
Change in body fat, % [a]	−1.9	(0.7)	−1.5	(0.7)	−0.5	(−2.4, 1.5)	
Waist circumference, cm	−5.2	(0.6)	−4.7	(0.6)	−0.5	(−2.3, 1.2)	
Hip circumference, cm	−5.5	(0.9)	−4.6	(0.9)	−1.0	(−3.6, 1.7)	
Waist-hip ratio	0.01	(0.00)	0.01	(0.00)	0.00	(−0.02, 0.02)	
SBP, mmHg	−2.5	(1.5)	−7.2	(1.5)	4.7	(0.5, 8.9)	
DBP, mmHg	−3.2	(1.0)	−3.1	(1.0)	−0.1	(−2.8, 2.6)	
Fat-responders	**High-fat diet (n=44)**		**High-carbohydrate diet (n=41)**				
	**Mean**	**(SD)**	**Mean**	**(SD)**	**Raw difference (95% CI)**		***p*-value**
Weight change, kg	−5.4	(0.6)	−5.2	(0.7)	−0.2	(−2.1, 1.6)	0.815
Weight change, %	−5.8	(0.7)	−5.7	(0.7)	−0.2	(−2.2, 1.7)	0.859
Change in body fat, % [b]	−1.6	(0.8)	−2.5	(0.8)	0.9	(−1.3, 3.0)	
Waist circumference, cm	−5.3	(0.7)	−4.7	(0.8)	−0.6	(−2.7, 1.5)	
Hip circumference, cm	−4.7	(0.8)	−4.9	(0.9)	0.2	(−2.2, 2.6)	
Waist-hip ratio	0.00	(0.00)	0.00	(0.00)	−0.01	(−0.03, 0.02)	
SBP, mmHg	−2.3	(1.8)	−9.2	(1.8)	6.9	(1.8, 12.0)	
DBP, mmHg	−3.6	(1.2)	−3.1	(1.2)	−0.5	(−3.9, 3.0)	

Carbohydrate-responders	**High-carbohydrate diet (n=16)**		**High-fat diet (n=21)**				
	**Mean**	**(SD)**	**Mean**	**(SD)**	**Raw difference (95% CI)**		***p*-value**
Weight change, kg	−5.0	(1.0)	−3.7	(0.9)	−1.3	(−4.1, 1.5)	0.344
Weight change, %	−5.5	(1.2)	−4.2	(1.1)	−1.2	(−4.3, 1.9)	0.429
Change in body fat, % [c]	−2.8	(1.5)	0.5	(1.3)	−3.4	(−7.4, 0.7)	
Waist circumference, cm	−5.0	(1.3)	−4.7	(1.1)	−0.3	(−3.8, 3.3)	
Hip circumference, cm	−7.8	(2.6)	−3.9	(2.3)	−3.9	(−10.9, 3.1)	
Waist-hip ratio	0.00	(0.00)	0.00	(0.00)	0.03	(−0.01, 0.08)	
SBP, mmHg	−3.0	(2.8)	−3.3	(2.5)	0.3	(−7.3, 7.9)	
DBP, mmHg	−2.2	(1.6)	−3.0	(1.4)	0.9	(−3.5, 5.3)	

Mixed-effect model for all data. Bold font indicates a significant difference between groups ($p<0.05$, 2-sided).

[a] Data available for 58 of 60 participants (genotype-concordant diet) and 60 of 62 participants (genotype-discordant diet).

[b] Data available for 42 of 44 participants (high-fat diet) and 40 of 41 participants (high-carbohydrate diet).

[c] Data available for 16 of 16 participants (high-carbohydrate diet) and 20 of 21 participants (high-fat diet).

Abbreviations: CI, confidence interval; DBP, diastolic blood pressure; SBP, systolic blood pressure; SD, standard deviation.

Supplementary Table 6. Changes in food cravings (via the Food Craving Inventory) during the 12-week intervention in those assigned to a diet concordant vs. discordant with the genotype (including raw difference).

All participants	**Genotype-concordant diet (n=60)**		**Genotype-discordant diet (n=62)**			
	**Mean**	**(SD)**	**Mean**	**(SD)**	**Raw difference (95% CI)**	
High fats [a]	−0.2	(0.1)	−0.3	(0.2)	0.1	(−0.1, 0.4)
Sweets [b]	−0.3	(0.1)	−0.5	(0.1)	0.2	(−0.1, 0.4)
Carbohydrates/Starches [c]	0.0	(0.1)	−0.3	(0.1)	0.3	(0.0, 0.5)
Fast-food fats [d]	−0.3	(0.1)	−0.4	(0.1)	0.1	(−0.2, 0.4)
Fruits and vegetables [e]	0.0	(0.1)	−0.2	(0.1)	0.2	(−0.1, 0.5)
Total cravings [f]	−0.2	(0.1)	−0.3	(0.1)	0.2	(−0.1, 0.4)
Fat-responders	**High-fat diet (n=44)**		**High-carbohydrate diet (n=41)**			
	**Mean**	**(SD)**	**Mean**	**(SD)**	**Raw difference (95% CI)**	
High fats [a]	−0.3	(0.1)	−0.2	(0.1)	0.0	(−0.3, 0.3)
Sweets [b]	−0.3	(0.1)	−0.5	(0.1)	0.2	(−0.1, 0.5)
Carbohydrates/Starches [c]	−0.1	(0.1)	−0.1	(0.1)	0.0	(−0.3, 0.4)
Fast-food fats [d]	−0.3	(0.1)	−0.2	(0.1)	−0.1	(−0.4, 0.3)
Fruits and vegetables [e]	−0.1	(0.1)	−0.2	(0.1)	0.1	(−0.3, 0.5)
Total cravings [f]	−0.2	(0.1)	−0.2	(0.1)	0.0	(−0.3, 0.3)

Carbohydrate-responders	High-carbohydrate diet (n=16)		High-fat diet (n=21)			
	**Mean**	**(SD)**	**Mean**	**(SD)**	**Raw difference (95% CI)**	
High fats [a]	−0.1	(0.1)	−0.6	(0.1)	0.5	(0.1, 0.9)
Sweets [b]	−0.2	(0.1)	−0.4	(0.1)	0.2	(−0.2, 0.6)
Carbohydrates/Starches [c]	0.2	(0.1)	−0.6	(0.1)	0.7	(0.4, 1.1)
Fast-food fats [d]	−0.1	(0.2)	−0.6	(0.2)	0.5	(0.0, 1.0)
Fruits and vegetables [e]	0.3	(0.2)	−0.3	(0.2)	0.6	(0.1, 1.1)
Total cravings [f]	0.0	(0.1)	−0.5	(0.1)	0.5	(0.2, 0.9)

Bold font indicates a significant difference between groups (p<0.05).

[a] Data available for 55 of 60 participants (genotype-concordant diet) and 60 of 62 participants (genotype-discordant diet). In fat-responders, data available for 41 of 44 participants (high-fat diet) and 40 of 41 participants (high-carbohydrate diet). In carbohydrate-responders, data available for 14 of 16 participants (high-carbohydrate diet) and 20 of 21 participants (high-fat diet).

[b] Data available for 59 of 60 participants (genotype-concordant diet) and 60 of 62 participants (genotype-discordant diet). In fat-responders, data available for 43 of 44 participants (high-fat diet) and 40 of 41 participants (high-carbohydrate diet). In carbohydrate-responders, data available for 16 of 16 participants (high-carbohydrate diet) and 20 of 21 participants (high-fat diet).

[c] Data available for 59 of 60 participants (genotype-concordant diet) and 61 of 62 participants (genotype-discordant diet). In fat-responders, data available for 44 of 44 participants (high-fat diet) and 40 of 41 participants (high-carbohydrate diet). In carbohydrate-responders, data available for 15 of 16 participants (high-carbohydrate diet) and 21 of 21 participants (high-fat diet).

[d] Data available for 58 of 60 participants (genotype-concordant diet) and 61 of 62 participants (genotype-discordant diet). In fat-responders, data available for 43 of 44 participants (high-fat diet) and 41 of 41 participants (high-carbohydrate diet). In carbohydrate-responders, data available for 15 of 16 participants (high-carbohydrate diet) and 20 of 21 participants (high-fat diet).

[e] Data available for 58 of 60 participants (genotype-concordant diet) and 60 of 62 participants (genotype-discordant diet). In fat-responders, data available for 43 of 44 participants (high-fat diet) and 40 of 41 participants (high-carbohydrate diet). In carbohydrate-responders, data available for 15 of 16 participants (high-carbohydrate diet) and 20 of 21 participants (high-fat diet).

[f] Data available for 54 of 60 participants (genotype-concordant diet) and 57 of 62 participants (genotype-discordant diet). In fat-responders, data available for 41 of 44 participants (high-fat diet) and 38 of 41 participants (high-carbohydrate diet). In carbohydrate-responders, data available for 13 of 16 participants (high-carbohydrate diet) and 19 of 21 participants (high-fat diet).

Abbreviations: CI, confidence interval; SD, standard deviation.

Supplementary Table 7. Change in restraint, disinhibition, and hunger and in food preferences during the 12-week intervention in those assigned to a diet concordant vs. discordant with the genotype (including raw difference).

All participants	**Genotype-concordant diet (n=60)**		**Genotype-discordant diet (n=62)**			
	**Mean**	**(SD)**	**Mean**	**(SD)**	**Raw difference (95% CI)**	
Restraint (EI) [a]	3.1	(0.5)	2.7	(0.5)	0.4	(−1.1, 1.9)
Disinhibition (EI) [b]	1.0	(0.4)	−0.9	(0.4)	0.0	(−1.0, 0.9)
Hunger (EI) [c]	−0.5	(0.3)	−1.0	(0.3)	0.5	(−0.4, 1.4)
HF/HS (FPQ)	−0.4	(0.2)	−0.4	(0.2)	0.0	(−0.5, 0.5)
LF/HS (FPQ)	−0.3	(0.2)	−0.3	(0.1)	0.1	(−0.4, 0.5)
HF/HCCHO (FPQ)	−0.5	(0.2)	−0.5	(0.2)	0.0	(−0.4, 0.5)
LF/HCCHO (FPQ)	−0.3	(0.2)	−0.2	(0.1)	−0.1	(−0.5, 0.4)
HF/LCHO/HP (FPQ)	−0.6	(0.2)	−0.6	(0.2)	0.0	(−0.5, 0.4)
LF/LCHO/HP (FPQ)	−0.3	(0.1)	−0.3	(0.1)	0.0	(−0.4, 0.5)
Fat-responders	**High-fat diet (n=44)**		**High-carbohydrate diet (n=41)**			
	**Mean**	**(SD)**	**Mean**	**(SD)**	**Raw difference (95% CI)**	
Restraint (EI) [a]	3.5	(0.7)	2.7	(0.7)	0.8	(−1.2, 2.8)
Disinhibition (EI) [b]	−1.2	(0.4)	−0.9	(0.5)	−0.4	(−1.6, 0.9)
Hunger (EI) [c]	−0.8	(0.4)	−1.2	(0.4)	0.4	(−0.8, 1.5)
HF/HS (FPQ)	−0.4	(0.2)	−0.5	(0.2)	0.1	(−0.6, 0.7)
LF/HS (FPQ)	−0.3	(0.2)	−0.3	(0.2)	0.0	(−0.6, 0.5)
HF/HCCHO (FPQ)	−0.5	(0.2)	−0.4	(0.2)	−0.1	(−0.6, 0.5)
LF/HCCHO (FPQ)	−0.4	(0.2)	−0.2	(0.2)	−0.2	(−0.7, 0.4)
HF/LCHO/HP (FPQ)	−0.5	(0.2)	−0.6	(0.2)	0.1	(−0.5, 0.7)
LF/LCHO/HP (FPQ)	−0.3	(0.2)	−0.4	(0.2)	0.1	(−0.4, 0.7)

Carbohydrate-responders	**High-carbohydrate diet (n=16)**		**High-fat diet (n=21)**			
	**Mean**	**(SD)**	**Mean**	**(SD)**	**Raw difference (95% CI)**	
Restraint (EI) [a]	2.0	(0.7)	2.7	(0.6)	−0.7	(−2.6, 1.2)
Disinhibition (EI) [b]	−0.2	(0.6)	−1.0	(0.5)	0.8	(−0.8, 2.4)
Hunger (EI) [c]	0.4	(0.5)	−0.7	(0.5)	1.0	(−0.4, 2.4)
HF/HS (FPQ)	−0.3	(0.3)	−0.2	(0.3)	−0.1	(−1.0, 0.7)
LF/HS (FPQ)	0.0	(0.2)	−0.3	(0.2)	0.3	(−0.3, 0.9)
HF/HCCHO (FPQ)	−0.4	(0.3)	−0.6	(0.3)	0.2	(−0.6, 1.0)
LF/HCCHO (FPQ)	0.0	(0.2)	−0.3	(0.2)	0.3	(−0.4, 0.9)
HF/LCHO/HP (FPQ)	−0.7	(0.3)	−0.5	(0.2)	−0.2	(−1.0, 0.5)
LF/LCHO/HP (FPQ)	−0.4	(0.2)	−0.2	(0.2)	−0.2	(−0.8, 0.4)

[a] Data available for 46 of 60 participants (genotype-concordant diet) and 47 of 62 participants (genotype-discordant diet). In fat-responders, data available for 34 of 44 participants (high-fat diet) and 29 of 41 participants (high-carbohydrate diet). In carbohydrate-responders, data available for 12 of 16 participants (high-carbohydrate diet) and 18 of 21 participants (high-fat diet).

[b] Data available for 49 of 60 participants (genotype-concordant diet) and 49 of 62 participants (genotype-discordant diet). In fat-responders, data available for 37 of 44 participants (high-fat diet) and 31 of 41 participants (high-carbohydrate diet). In carbohydrate-responders, data available for 12 of 16 participants (high-carbohydrate diet) and 18 of 21 participants (high-fat diet).

[c] Data available for 51 of 60 participants (genotype-concordant diet) and 51 of 62 participants (genotype-discordant diet). In fat-responders, data available for 37 of 44 participants (high-fat diet) and 33 of 41 participants (high-carbohydrate diet). In carbohydrate-responders, data available for 14 of 16 participants (high-carbohydrate diet) and 18 of 21 participants (high-fat diet).

Abbreviations: CI, confidence interval; EI, Eating Inventory; FPQ, Food Preference Questionnaire; HF/HS, high fat/high simple sugar; LF/HS, low fat/high simple sugar; HF/HCCHO, high fat/high complex carbohydrate; LF/HCCHO, low fat/high complex carbohydrate; HF/LCHO/HP, high fat/low carbohydrate/high protein; LF/LCHO/HP, low fat/low carbohydrate/high protein; SD, standard deviation.

Supplementary Table 8. Change in items of the Diet Personalization Survey during the 12-week intervention as well as intervention satisfaction (post-intervention) in those assigned to a diet concordant vs. discordant with the genotype (including raw difference).

All participants	Genotype-concordant diet (n=60)	Genotype-discordant diet (n=62)	
	Mean [a] (SD)	Mean [a] (SD)	Raw difference (95% CI)
**Diet Personalization Survey**			
The assigned diet…			
… fits my typical eating habits	−0.2 (0.3)	0.1 (0.3)	−0.3 (−1.2, 0.6)
… fits my lifestyle	0.0 (0.3)	−0.2 (0.3)	0.2 (−0.7, 1.1)
… makes it easier to lose weight	−0.6 (0.3)	−0.6 (0.3)	0.1 (−0.8, 0.8)
I am confident that I can…			
… successfully lose weight on the assigned diet	−0.6 (0.3)	−0.5 (0.3)	−0.1 (−0.8, 0.7)
… follow the assigned diet	−0.8 (0.2)	−0.6 (0.2)	−0.3 (−0.9, 0.4)
**Intervention Satisfaction Survey**	**Mean [c] (SD)**	**Mean [c] (SD)**	**Raw difference (95% CI)**
I am satisfied with…			
… the group format	6.8 (0.2)	7.7 (0.2)	−0.5 (−1.0, 0.1)
… the support from interventionists	7.4 (0.2)	7.3 (0.2)	0.1 (−0.4, 0.5)
… the intervention materials	7.0 (0.2)	7.1 (0.2)	−0.1 (−0.6, 0.4)
… the support from other participants	6.6 (0.2)	6.6 (0.2)	−0.1 (−0.7, 0.6)
… the amount of food in my meal plan	6.1 (0.3)	5.9 (0.3)	0.1 (−0.6, 0.8)
… the macronutrient content in my meal plan	6.1 (0.2)	5.8 (0.2)	0.3 (−0.4, 1.0)
… my progress toward weight management	6.1 (0.3)	5.8 (0.3)	0.3 (−0.5, 1.0)
… the degree to which the diet helped manage hunger	6.4 (0.3)	5.9 (0.3)	0.5 (−0.3, 1.2)

Fat-responders	High-fat diet (n=44)	High-carbohydrate diet (n=41)	
	**Mean [a] (SD)**	**Mean [a] (SD)**	**Raw difference (95% CI)**
**Diet Personalization Survey**			
The assigned diet…			
… fits my typical eating habits	−0.3 (0.3)	0.4 (0.4)	−0.6 (−1.6, 0.4)
… fits my lifestyle	−0.1 (0.4)	0.0 (0.4)	−0.1 (−1.2, 0.9)
… makes it easier to lose weight	−0.7 (0.4)	−0.6 (0.4)	−0.1 (−1.1, 0.9)
I am confident that I can…			
… successfully lose weight on the assigned diet	−0.7 (0.3)	−0.6 (0.3)	−0.1 (−1.1, 0.8)
… follow the assigned diet	−0.9 (0.3)	−0.5 (0.3)	−0.4 (−1.2, 0.5)
**Intervention Satisfaction Survey**	**Mean [b] (SD)**	**Mean [b] (SD)**	**Raw difference (95% CI)**
I am satisfied with…			
… the group format	6.7 (0.2)	7.3 (0.2)	−0.6 (−1.2, 0.1)
… the support from interventionists	7.3 (0.2)	7.4 (0.2)	−0.1 (−0.7, 0.4)
… the intervention materials	6.8 (0.2)	7.1 (0.2)	−0.3 (−0.9, 0.3)
… the support from other participants	6.3 (0.3)	6.7 (0.3)	−0.4 (−1.2, 0.4)
… the amount of food in my meal plan	6.0 (0.3)	5.9 (0.3)	0.0 (−0.9, 0.9)
… the macronutrient content in my meal plan	6.1 (0.3)	5.8 (0.3)	0.3 (−0.5, 1.2)
… my progress toward weight management	6.2 (0.3)	5.8 (0.3)	0.4 (−0.5, 1.2)
… the degree to which the diet helped manage hunger	6.5 (0.3)	5.9 (0.3)	0.6 (−0.3, 1.4)

Carbohydrate-responders	High-carbohydrate diet (n=16)	High-fat diet (n=21)	
	**Mean [a] (SD)**	**Mean [a] (SD)**	**Raw difference (95% CI)**
**Diet Personalization Survey**			
The assigned diet…			
… fits my typical eating habits	0.1 (0.7)	−0.3 (0.6)	0.4 (−1.4, 2.2)
… fits my lifestyle	0.4 (0.6)	−0.6 (0.5)	1.0 (−0.6, 2.6)
… makes it easier to lose weight	−0.2 (0.5)	−0.6 (0.4)	0.4 (−0.9, 1.7)
I am confident that I can…			
… successfully lose weight on the assigned diet	−0.2 (0.5)	−0.4 (0.5)	0.2 (−1.3, 1.6)
… follow the assigned diet	−0.6 (0.4)	−0.6 (0.4)	0.0 (−1.1, 1.1)
**Intervention Satisfaction Survey**	**Mean [b] (SD)**	**Mean [b] (SD)**	**Raw difference (95% CI)**
I am satisfied with…			
… the group format	7.1 (0.4)	7.3 (0.4)	−0.2 (−1.4, 0.9)
… the support from interventionists	7.6 (0.4)	7.1 (0.3)	0.5 (−0.5, 1.5)
… the intervention materials	7.5 (0.3)	7.1 (0.3)	0.4 (−0.5, 1.3)
… the support from other participants	7.3 (0.4)	6.6 (0.4)	0.8 (−0.3, 1.9)
… the amount of food in my meal plan	6.3 (0.5)	5.9 (0.4)	0.4 (−0.8, 1.6)
… the macronutrient content in my meal plan	6.1 (0.5)	5.8 (0.4)	0.4 (−0.9, 1.7)
… my progress toward weight management	5.8 (0.5)	5.8 (0.5)	−0.1 (−1.5, 1.4)
… the degree to which the diet helped manage hunger	6.2 (0.6)	5.9 (0.5)	0.3 (−1.2, 1.8)

[a] Mean change during the 12-week intervention.

[b] Mean post-intervention value. The Intervention Satisfaction Survey was only assessed at Week 12.

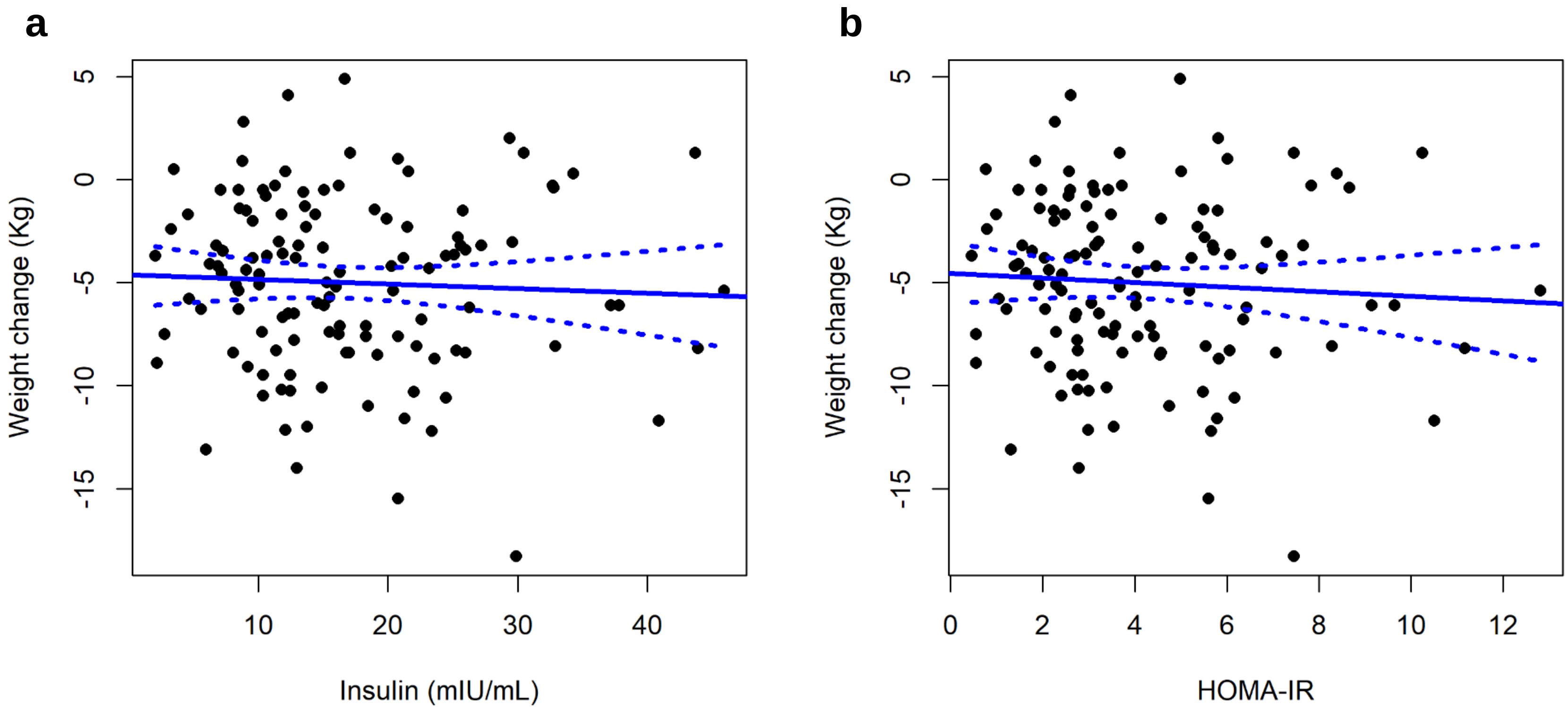

Supplementary Figure 1. Association between (a) fasting insulin at baseline and 12-week weight change (β=−0.036 [95% CI: −0.125, 0.053, *p*=0.43]) and (b) HOMA-IR at baseline and 12-week weight change (β=−0.165 [95% CI: −0.505, 0.175, *p*=0.34]). Linear mixed model (2-sided), adjusted for diet group, sex, race, and baseline weight. The centers are the estimated effects of baseline insulin and HOMA-IR, as well as the interaction terms, on weight loss, sex, race, diet group, and baseline weight.

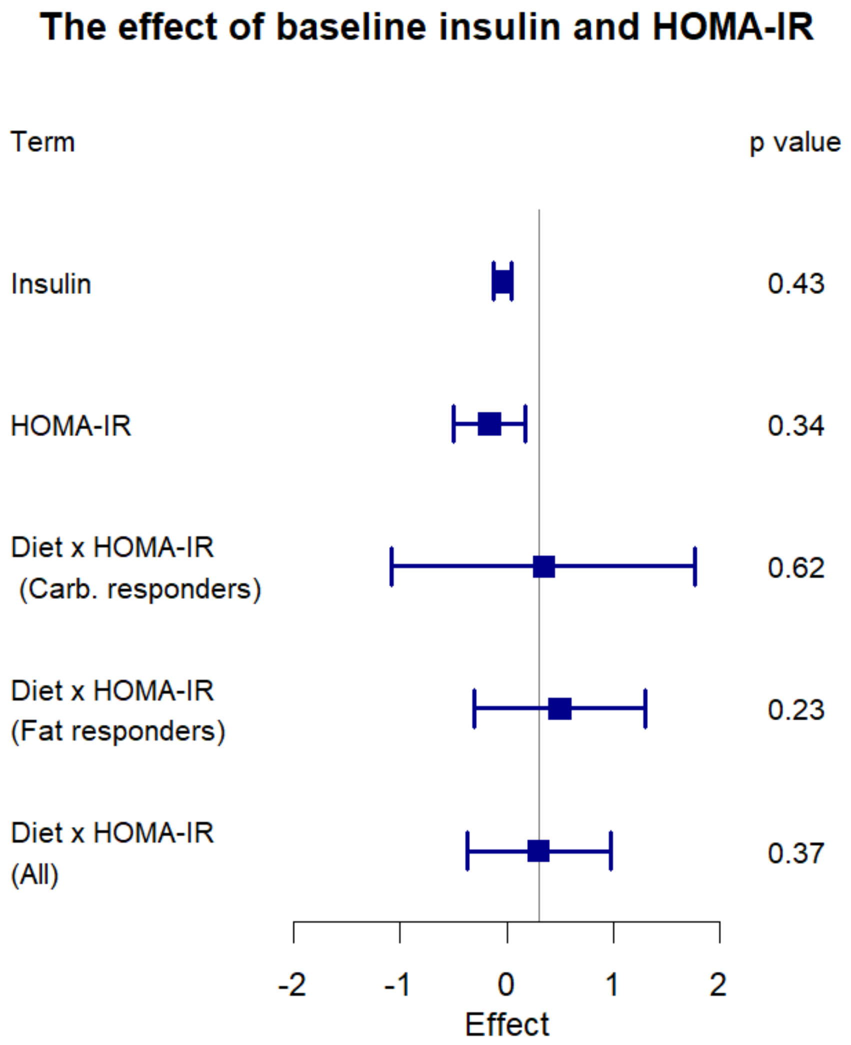

Supplementary Figure 2. Forest plot of the diet × baseline HOMA-IR interaction on weight change (all participants n=122, carbohydrate-responders n=37, fat-responders n=85). Linear mixed model (2-sided), adjusted for diet group, sex, and race. The error bars represent the 95% confidence intervals for the effect estimates.

Supplementary Table 9. Attendance by diet and genotype group, among completed subjects.

		Fat-responders		Carbohydrate-responders		
		**High-fat diet**	**High-carbohydrate diet**	**High-fat diet**	**High-carbohydrate diet**	**Total adherence**
**Session**	**Attended**	**n (%)**	**n (%)**	**n (%)**	**n (%)**	**n (%)**
**Week 1**	No	0 (0.0)	1 (2.6)	0 (0.0)	0 (0.0)	1 (0.9)
	Yes	36 (100.0)	38 (97.4)	20 (100.0)	16 (100.0)	110 (99.1)
**Week 2**	No	2 (5.6)	6 (15.4)	0 (0.0)	0 (0.0)	8 (7.2)
	Yes	34 (94.4)	33 (84.6)	20 (100.0)	16 (100.0)	103 (92.8)
**Week 3**	No	0 (0.0)	4 (10.3)	0 (0.0)	0 (0.0)	4 (3.6)
	Yes	36 (100.0)	35 (89.7)	20 (100.0)	16 (100.0)	107 (96.4)
**Week 4**	No	0 (0.0)	4 (10.3)	1 (5.0)	0 (0.0)	5 (4.5)
	Yes	36 (100.0)	35 (89.7)	19 (95.0)	16 (100.0)	106 (95.5)
**Week 5**	No	0 (0.0)	4 (10.3)	0 (0.0)	0 (0.0)	4 (3.6)
	Yes	36 (100.0)	35 (89.7)	20 (100.0)	16 (100.0)	107 (96.4)
**Week 6**	No	0 (0.0)	3 (7.7)	0 (0.0)	0 (0.0)	3 (2.7)
	Yes	36 (100.0)	36 (92.3)	20 (100.0)	16 (100.0)	108 (97.3)
**Week 7**	No	2 (5.6)	3 (7.7)	0 (0.0)	0 (0.0)	5 (4.5)
	Yes	34 (94.4)	36 (92.3)	20 (100.0)	16 (100.0)	106 (95.5)
**Week 8**	No	0 (0.0)	2 (5.1)	1 (5.0)	0 (0.0)	3 (2.7)
	Yes	36 (100.0)	37 (94.9)	19 (95.0)	16 (100.0)	108 (97.3)
**Week 9**	No	1 (2.8)	4 (10.3)	1 (5.0)	1 (6.2)	7 (6.3)
	Yes	35 (97.2)	35 (89.7)	19 (95.0)	15 (93.8)	104 (93.7)
**Week 10**	No	2 (5.6)	6 (15.4)	0 (0.0)	1 (6.2)	9 (8.1)
	Yes	34 (94.4)	33 (84.6)	20 (100.0)	15 (93.8)	102 (91.9)
**Week 11**	No	0 (0.0)	5 (12.8)	1 (5.0)	0 (0.0)	6 (5.5)
	Yes	36 (100.0)	34 (87.2)	19 (95.0)	16 (100.0)	104 (94.5)
**Week 12**	No	2 (5.6)	4 (10.3)	0 (0.0)	0 (0.0)	6 (5.4)
	Yes	34 (94.4)	35 (89.7)	20 (100.0)	16 (100.0)	105 (94.6)

Attendance data are not available for all subjects.

SUPPLEMENTARY REFERENCES

1. Lai, C.-Q. *et al.* Epigenomics and metabolomics reveal the mechanism of the APOA2-saturated fat intake interaction affecting obesity. *Am. J. Clin. Nutr.* **108**, 188–200 (2018).
2. Smith, C. E. *et al.* Apolipoprotein A2 polymorphism interacts with intakes of dairy foods to influence body weight in 2 U.S. populations. *J. Nutr.* **143**, 1865–1871 (2013).
3. Corella, D. *et al.* Association between the APOA2 promoter polymorphism and body weight in Mediterranean and Asian populations: replication of a gene-saturated fat interaction. *Int. J. Obes.* **35**, 666–675 (2011).
4. Corella, D. *et al.* APOA2, dietary fat, and body mass index: replication of a gene-diet interaction in 3 independent populations. *Arch. Intern. Med.* **169**, 1897–1906 (2009).
5. Corella, D. *et al.* The -256T>C polymorphism in the apolipoprotein A-II gene promoter is associated with body mass index and food intake in the genetics of lipid lowering drugs and diet network study. *Clin. Chem.* **53**, 1144–1152 (2007).
6. Stunkard, A. J. & Messick, S. The three-factor eating questionnaire to measure dietary restraint, disinhibition and hunger. *J. Psychosom. Res.* **29**, 71–83 (1985).
7. White, M. A., Whisenhunt, B. L., Williamson, D. A., Greenway, F. L. & Netemeyer, R. G. Development and validation of the food-craving inventory. *Obes. Res.* **10**, 107–114 (2002).
8. Geiselman, P. J. *et al.* Reliability and validity of a macronutrient self-selection paradigm and a food preference questionnaire. *Physiol. Behav.* **63**, 919–928 (1998).
