# Supplementary material for: The Personalized Nutrition Study (POINTS): evaluation of a genetically informed weight loss approach, a Randomized Clinical Trial
Source: Nat Commun. 2023 Oct 9;14:6321. doi: 10.1038/s41467-023-41969-1 (PMC10562431; doi:10.1038/s41467-023-41969-1)
Supplement: Supplementary file 3 — Reporting Summary [file 41467_2023_41969_MOESM3_ESM.pdf]

nature portfolio

Corresponding author(s): Christoph Höchsmann

Last updated by author(s): Sep 14, 2023

Reporting Summary

Nature Portfolio wishes to improve the reproducibility of the work that we publish. This form provides structure for consistency and transparency in reporting. For further information on Nature Portfolio policies, see our Editorial Policies and the Editorial Policy Checklist.

Statistics

For all statistical analyses, confirm that the following items are present in the figure legend, table legend, main text, or Methods section.

n/a	Confirmed	
☐	☒	The exact sample size (*n*) for each experimental group/condition, given as a discrete number and unit of measurement
☐	☒	A statement on whether measurements were taken from distinct samples or whether the same sample was measured repeatedly
☐	☒	The statistical test(s) used AND whether they are one- or two-sided<br>*Only common tests should be described solely by name; describe more complex techniques in the Methods section.*
☐	☒	A description of all covariates tested
☐	☒	A description of any assumptions or corrections, such as tests of normality and adjustment for multiple comparisons
☐	☒	A full description of the statistical parameters including central tendency (e.g. means) or other basic estimates (e.g. regression coefficient) AND variation (e.g. standard deviation) or associated estimates of uncertainty (e.g. confidence intervals)
☐	☒	For null hypothesis testing, the test statistic (e.g. *F*, *t*, *r*) with confidence intervals, effect sizes, degrees of freedom and *P* value noted<br>*Give P values as exact values whenever suitable.*
☒	☐	For Bayesian analysis, information on the choice of priors and Markov chain Monte Carlo settings
☒	☐	For hierarchical and complex designs, identification of the appropriate level for tests and full reporting of outcomes
☒	☐	Estimates of effect sizes (e.g. Cohen's *d*, Pearson's *r*), indicating how they were calculated

Our web collection on statistics for biologists contains articles on many of the points above.

Software and code

Policy information about availability of computer code

Data collection	Data for surveys in this study were collected and managed using REDCap tools.. No other software was used for data collection.
Data analysis	All analyses were conducted using SAS (Windows version 9.4; SAS Institute, Cary, NC) and the statistical program R version 4.0.2 (https://cran.r-project.org/). We did not use any custom code/function that is not available publicly. The code used for the statistical analyses is available via figshare (https://doi.org/10.6084/m9.figshare.24121110).

For manuscripts utilizing custom algorithms or software that are central to the research but not yet described in published literature, software must be made available to editors and reviewers. We strongly encourage code deposition in a community repository (e.g. GitHub). See the Nature Portfolio guidelines for submitting code & software for further information.

Data

Policy information about availability of data

All manuscripts must include a data availability statement. This statement should provide the following information, where applicable:
- Accession codes, unique identifiers, or web links for publicly available datasets
- A description of any restrictions on data availability
- For clinical datasets or third party data, please ensure that the statement adheres to our policy

The complete de-identified data generated in this study, including all outcome measures and the study protocol have been deposited (open access, CC BY 4.0) in the figshare repository and are available at the following link: https://doi.org/10.6084/m9.figshare.24121110. Source data are provided with this paper.

Human research participants

Policy information about studies involving human research participants and Sex and Gender in Research.

Reporting on sex and gender	Sex was included as a covariate in our models. We did not perform subgroup analyses by sex or gender.
Population characteristics	Participants were 54.4 (SD: 13.2) years old, with no difference between groups. Race was included as a covariate in our models, and participants were predominantly White (68%) and Black/African American (29.5%), with 2.5% being other races.
Recruitment	Participants were recruited from the community. Potential subjects were identified via a large database of previous study participants along with the use of web, print, and media advertising. Pennington Biomedical Research Center's (PBRC) Recruitment and Advertising Cores created and implemented individualized, trial-specific advertising and awareness campaigns, including mass media, traditional advertising, and novel methods including social media, digital and email marketing. All advertising and awareness were approved by our on-site IRB to ensure ethical and disclosure standards were met. The PBRC database was also utilized to find potential subjects for this study. We see no selection bias, outside of the fact that the study recruited volunteers, who may be different from other people in the target population.
Ethics oversight	The study was approved by the institutional review board and conducted at Pennington Biomedical Research Center (Baton Rouge, LA, USA)

Note that full information on the approval of the study protocol must also be provided in the manuscript.

Field-specific reporting

Please select the one below that is the best fit for your research. If you are not sure, read the appropriate sections before making your selection.

☐ Life sciences ☒ Behavioural & social sciences ☐ Ecological, evolutionary & environmental sciences

For a reference copy of the document with all sections, see nature.com/documents/nr-reporting-summary-flat.pdf

Behavioural & social sciences study design

All studies must disclose on these points even when the disclosure is negative.

Study description	Randomized Clinical Trial, quantitative data
Research sample	Participants were recruited from the community. Eligible participants were men or women, 18-75 years old, had a BMI of 27.0-47.5 kg/m2, and had completed or were willing to complete a genealogy test (e.g., Ancestry, 23andMe) and to share the raw data with the investigators. Finally, a genetic profile indicating a predisposition to respond favorably to either a high-carbohydrate or high-fat WL diet based on specific SNPs (see below) was required. Exclusion criteria included smoking, weight change ≥10 lbs. in the last 3 months, being pregnant or breastfeeding, conditions, diseases, or medications that affect body weight or metabolism or could affect risk or study completion, and a genotype indicating a predisposition to respond favorably to neither or both of the specified diets. We estimated that approximately 1/3 of people would be fat-responders, 1/3 carbohydrate-responders, and 1/3 would respond favorably to neither or both of the specified diets. Baseline characteristics of all 122 included participants (84% women, 68% White) were 54.4 (SD: 13.2) years of age and a BMI of 34.9 (SD: 5.1) kg/m2. The eligibility criteria were designed to be as inclusive as possible and recruit a sample that is representative of weight loss treatment seeking adults. We expect that the sample is representative of the target population, with the exception of participants having volunteered for the project (vs. being randomly selected from the target population, which is impossible and unethical).
Sampling strategy	The PBRC biostatistics department created the randomization sequence using SAS 9.4 statistical software for Windows (SAS Institute, Cary, NC) and uploaded it to REDCap (Research Electronic Data Capture). REDCap used strata for the inaction of genetic responder and gender. A 1:1 allocation using random block sizes of 2, 4, and 6 was used and adapted by the biostatistician during the study to ensure equal numbers of men and women and a similar BMI across diet groups and strata.<br><br>The present study planned to obtain data from up to 154 participants in total, and we aimed to complete 32 participants per group (128 participants in total) though we did not limit recruitment to achieve equal numbers of participants in each group. We hypothesized participants on a genotype-concordant diet would lose more weight than those on a genotype-discordant diet. Based on previous studies,49,50 we assumed a standard deviation for between-group differences in weight change of 2.8 kg. To detect a 2.0 kg difference in weight change between group 1 (fat-responders on a high-fat diet) and group 2 (fat-responders on a high-carbohydrate diet) or between group 3 (carbohydrate-responders on a high-fat diet) and group 4 (carbohydrate-responders on a high-carbohydrate diet), with the intended sample size and an alpha level of 0.05, the present study would have 80% power. Further, based on the same assumptions, the present study would have >95% power to test if WL differs between participants on a genotype-concordant diet (groups 1 and 4 combined) and those on a genotype-discordant diet (groups 2 and 3).
Data collection	Anthropometric data<br>Fasting body weight and waist and hip circumference were measured in the PBRC outpatient clinic. Body fat (%, via bioelectrical impedance analysis [BIA]; X-contact 365, Jawon Medical Co., Ltd, Seoul, South Korea) and blood pressure (after 5 minutes of seated

rest) were also measured in the PBRC outpatient clinic.

Fasting serum glucose and insulin
Fasting serum glucose and insulin were measured at W0 and HOMA-IR was used to quantify insulin resistance.

Appetitive traits, food cravings, and food preferences
Appetitive traits were measured with the Eating Inventory (EI), food cravings were measured with the Food Craving Inventory (FCI), and hedonic food preferences were measured with the Food Preference Questionnaire (FPQ).

Diet personalization and intervention satisfaction were assessed via self-developed questionnaires.

Outcome assessors were blind to diet assignment and genotype patterns. Participants were only informed of their genotype (carbohydrate- or fat-responder) once they completed the study.

All data were collected in the PBRC outpatient clinic by trained staff, with only the participant and the clinic staff present.

Timing	The study was conducted between February 2020 and December 2021.
Data exclusions	Seven participants were excluded from the analyses because they were incorrectly genotyped (n=5; removal from dataset suggested by IRB) or failed to provide weight data at W12 (n=2).
Non-participation	Of the 145 participants randomized, 16 were lost to follow-up (W12) and 129 completed the trial.
Randomization	The PBRC biostatistics department created the randomization sequence using SAS 9.4 statistical software for Windows (SAS Institute, Cary, NC) and uploaded it to REDCap (Research Electronic Data Capture). REDCap used strata for the inaction of genetic responder and gender. A 1:1 allocation using random block sizes of 2, 4, and 6 was used and adapted by the biostatistician during the study to ensure equal numbers of men and women and a similar BMI across diet groups and strata.

Reporting for specific materials, systems and methods

We require information from authors about some types of materials, experimental systems and methods used in many studies. Here, indicate whether each material, system or method listed is relevant to your study. If you are not sure if a list item applies to your research, read the appropriate section before selecting a response.

Materials & experimental systems

n/a	Involved in the study	
☒	☐	Antibodies
☒	☐	Eukaryotic cell lines
☒	☐	Palaeontology and archaeology
☒	☐	Animals and other organisms
☐	☒	Clinical data
☒	☐	Dual use research of concern

Methods

n/a	Involved in the study	
☒	☐	ChIP-seq
☒	☐	Flow cytometry
☒	☐	MRI-based neuroimaging

Clinical data

Policy information about clinical studies

All manuscripts should comply with the ICMJEguidelines for publication of clinical research and a completedCONSORT checklist must be included with all submissions.

Clinical trial registration	NCT04145466
Study protocol	uploaded with submission
Data collection	The study conducted at Pennington Biomedical Research Center (Baton Rouge, LA) between February 2020 and December 2021.
Outcomes	The primary outcome was weight change (kg) at 12 weeks. All other measures were pre-defined secondary endpoints (see study protocol and also NCT04145466. These were:<br><br>Waist and hip circumference (extensible tape measure)<br><br>Body fat (%, via bioelectrical impedance analysis [BIA]; X-contact 365, Jawon Medical Co., Ltd, Seoul, South Korea)<br><br>Blood pressure (sphygmomanometer, after 5 minutes of seated rest)<br><br>Fasting serum glucose and insulin by standard laboratory analysis of venous blood.<br><br>Appetitive traits (Eating Inventory), food cravings (Food Craving Inventory), and hedonic food preferences (Food Preference Questionnaire).<br><br>Diet personalization and intervention satisfaction were assessed via self-developed questionnaires.
